# Single‐cell RNA sequencing reveals heterogeneity and differential expression of decidual tissues during the peripartum period

**DOI:** 10.1111/cpr.12967

**Published:** 2020-12-09

**Authors:** Jingrui Huang, Qi Li, Qiaozhen Peng, Yingming Xie, Weinan Wang, Chenlin Pei, Yanhua Zhao, Rong Liu, Lihui Huang, Tieping Li, Liangqun Xie, Jiejie Zhang, Lei Dai, Jingfei Chen, Jingchi Sun, Weishe Zhang

**Affiliations:** ^1^ Department of Obstetrics Xiangya Hospital Central South University Changsha China; ^2^ Department of Obstetrics and Gynecology Wuhan Union Hospital Tongji Medical College Huazhong University of Science and Technology Wuhan China; ^3^ Department of Obstetrics and Gynecology Changsha Hospital for Maternal and Child Health Care Changsha China; ^4^ Hunan Engineering Research Center of Early Life Development and Disease Prevention Changsha China

**Keywords:** decidua, heterogeneity, labour onset, single‐cell sequencing, transcriptome

## Abstract

**Objectives:**

The decidua is a tissue that contacts both maternal and foetal components and is pivotal to labour onset due to its location. Due to the heterogeneity of decidual tissue, it is challenging to study its role in the peripartum period. Herein, we analysed the transcriptomes of peripartum decidua at single‐cell resolution.

**Materials and methods:**

Single‐cell RNA sequencing was performed for 29 231 decidual cells before and after delivery to characterize the transcriptomes.

**Results:**

Eight major cell types (including endothelial cells, fibroblasts) and subtypes of decidual stromal cells, extravillous trophoblasts and T cells were identified and found to have various functions. Compared with before delivery, the activation of decidual stromal cell, extravillous trophoblast and T‐cell subtypes to different degrees was observed after delivery. Furthermore, the activation involved multiple functions, such as cell proliferation, and several pathways, such as the activator protein 1 pathway. The results of pseudotemporal ordering showed differentiation of decidual stromal cell and extravillous trophoblast subtypes, suggesting inhomogeneity of these subgroups in decidualization (decidual stromal cell) and invasion (extravillous trophoblast).

**Conclusions:**

The peripartum decidual tissue is heterogeneous. This study revealed changes in the decidua and its components at single‐cell resolution; these findings provide a new perspective for the study of peripartum decidua.

## Graphical abstract

Single‐cell RNA sequencing was used to characterize the transcriptomes of decidua before and after delivery. Eight major cell types and sixteen clusters were identified and found to have various functions. The activation of various cell subtypes to different degrees was observed after delivery. These reveal the heterogeneity of peripartum decidual cells at single‐cell resolution

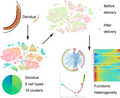

## 1 INTRODUCTION

Abnormal onset of labour is a direct cause of several adverse pregnancy events, especially preterm birth.^1^, ^2^, ^3^, ^4^ As a pregnancy‐specific tissue, the maternal‐foetal interface is essential for delivery.^5^, ^6^, ^7^, ^8^, ^9^ Decidua is one of the central tissues found within the interface that contacts both maternal and foetal components; it is a tissue with high heterogeneity. Decidua is the target of decidualization during implantation^10^ and plays an important role in labour onset.^11^

Various cell types in decidual tissue, such as decidual stromal cells and T cells, have been uncovered by classical cell morphology classification, flow cytometry and other methods. The recent development of single‐cell RNA sequencing has resulted in a more comprehensive understanding of tissue components. Published studies on the placenta in the first and second trimesters have revealed their role in preeclampsia and other pathological pregnancies.^12^, ^13^, ^14^ Studies on the third trimester have revealed insights on the differentiation of placental trophoblasts, placental cellular dynamics and the interactions between placental trophoblast cells and decidual stromal cells.^15^, ^16^ Importantly, these studies also found that the decidua was at the centre of intercellular signal transduction. These studies demonstrate the essential role of the decidua throughout pregnancy, as well as the importance of studying the cell types, cell ratios and cell heterogeneity of the decidua for understanding labour onset.

Here, we performed single‐cell RNA sequencing of the decidua before and after delivery, and identified the major cell populations and subpopulations. The functions of different cell types and subtypes during labour onset were evaluated by bioinformatics. The results revealed decidual cell changes during labour onset. Overall, the results from this study provide the basis for further research.

## 2 MATERIALS AND METHODS

### 2.1 Study population

All patients were diagnosed with singleton pregnancy in the Xiangya Hospital Central South University or Changsha Hospital for Maternal and Child Health Care between 1 October 2018 and 1 January 2019. The study protocol was approved by the Medical Ethics Committee of the Xiangya Hospital Central South University (2018081027) and Changsha Hospital for Maternal and Child Health Care Ethics Committee (2018810). Informed consent was obtained from all patients prior to data collection. All procedures involving human participants were performed in accordance with the ethical standards of the Institutional Research Committee, and the Helsinki Declaration and its later amendments or comparable ethical standards. The authors had no access to information that could identify individual participants during and after data collection. This study included six pregnant women. Of these, three had vaginal births and the remaining three had caesarean sections (without labour onset). The general information of all patients is shown in Table S1.

### 2.2 Decidual tissue dissociation

According to the general steps of making single‐cell suspension,^12^, ^13^, ^14^ for term pregnancy (labour onset), three decidual samples were obtained after delivery and washed in phosphate‐buffered saline (37°C). Similarly, three samples before delivery were obtained during cesarean section (without labour). Subsequently, the samples were dissociated with tweezers and scissors, and a single‐cell suspension was made after dissociation, digestion (collagenase Sigma‐Aldrich, and phosphate‐buffered saline, 37°C, 45 minutes) and filtration (Falcon 40‐μm cell strainer; Corning). Then, the cell viability assessment was performed before the next step.

### 2.3 Single‐cell cDNA library preparation and sequencing

This process was performed by CapitalBio Technology Corporation and in accordance with the manufacturer's and previous instructions.^17^ A Single Cell 5’ Library Gel Bead Kit was used to construct single‐cell RNA‐sequencing libraries according to the manufacturer's instructions. The library was sequenced using an Illumina NovaSeq 6000 sequencer with a depth of at least 100 000 reads per cell (performed by CapitalBio).

### 2.4 Single‐cell RNA‐sequencing data analysis

Cell Ranger 2.0.1 was used to analyse the data. The data from different samples were merged through a Cell Ranger aggr pipeline and normalized by equalizing the read depth. Principal component analysis and t‐distributed stochastic neighbour embedding were performed using the R t‐distributed stochastic neighbour embedding package of r software. According to the guide, output and presentation files were displayed and analysed by the Cell Browser 2.0.0 (10 × Genomics).

### 2.5 Gene ontology and pathway analysis

For the output gene list of each cluster, we analysed it through Metascape.^18^ KEGG pathway, gene ontology biological processes, reactome gene sets, canonical pathways and CORUM were used for the pathway and process enrichment analysis. Based on the accumulative hypergeometric distribution, the *P*‐values were calculated.

### 2.6 Pseudotemporal ordering analysis

We used the Monocle package (r) for pseudotemporal ordering analysis. Genes that met the following standards were selected: gene expressed in no less than 10 cells, average expression value greater than 0.5, *q* value of the differential expression analysis less than 0.01 and dispersion value greater than or equal to the expected dispersion value. After selection, the multidimensional space was reduced to two‐dimensional space, and then, the single cell was sorted (Monocle).

## 3 RESULTS

### 3.1 Construction of cell spectrum of decidual tissue during the peripartum period

**FIGURE 1:**
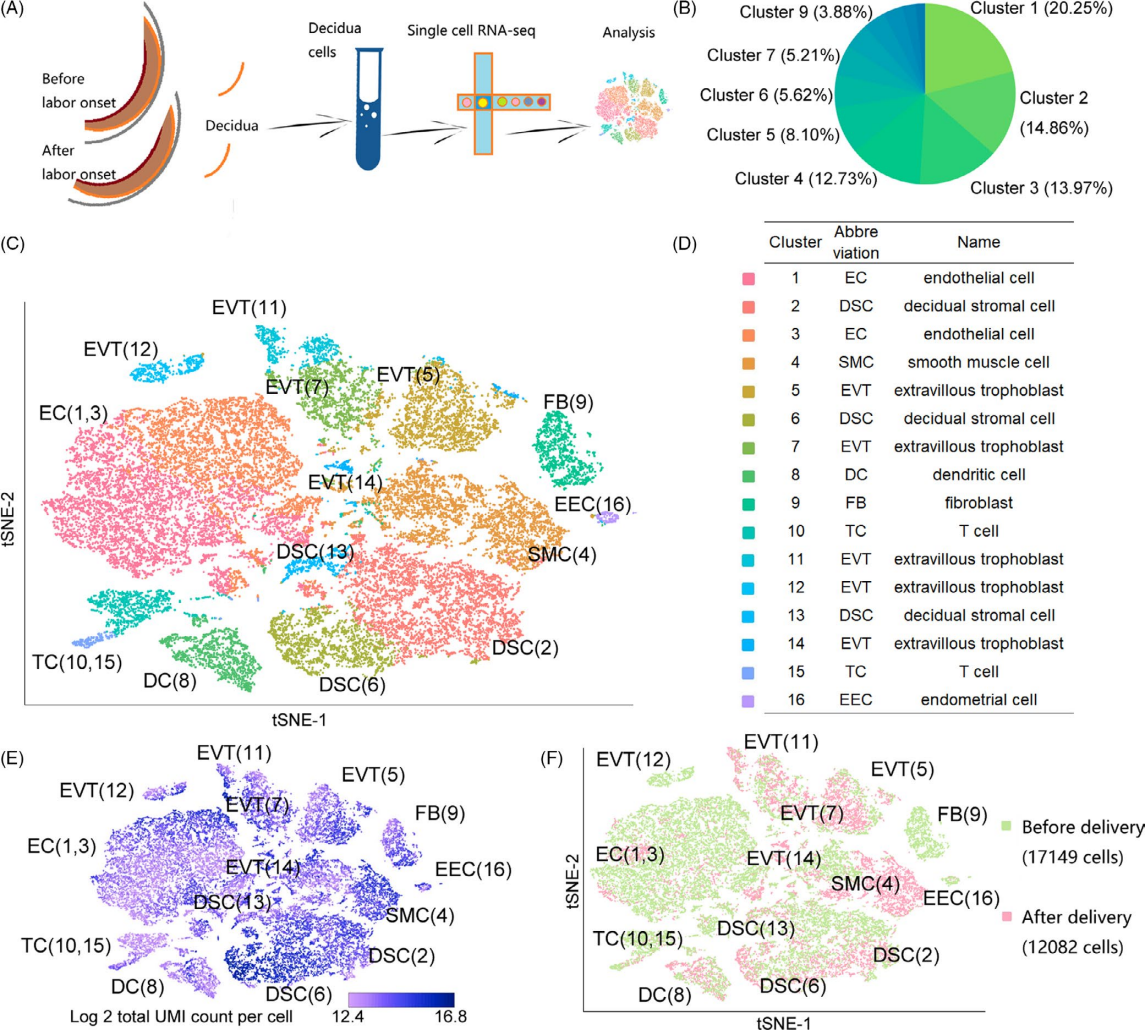
Single‐cell RNA‐sequencing profiling and 16 distinct decidual cell populations. A, Overview of the single‐cell RNA‐sequencing analysis workflow. B, Cell number proportion in each cluster. C, t‐SNE projection of 29 231 decidual cells (each point represents a single cell). The projection where cells that share similar transcriptome profiles are grouped by colours (16 distinct cell populations) representing unsupervised clustering results. D, Table showing the assigned identity of each cluster. E, Relative UMI value per cell. F, Distribution of cells in different states. Green represents the state before delivery, and red represents the state after delivery. t‐SNE, t‐distributed stochastic neighbour embedding; UMI, unique molecular identifier

All of the decidual tissue samples were collected for single‐cell sequencing (Figure 1A). A total of 29 231 cells were detected and divided into 16 cell clusters, with different relative unique molecular identifier value per cell (Figure 1B,C). A total of 17 149 cells (three samples) before labour onset and 12 082 cells (three samples) after delivery were analysed (Figure 1D). Using the Cell Marker database^19^ combined with published literature,^13^, ^14^, ^15^, ^20^, ^21^ eight cell types were identified (Figure 1E,F), including endothelial cells, decidual stromal cells, extravillous trophoblasts and T cells. In addition, smooth muscle cells were identified by *MSC*, *MYH11*, *FOXS1* and *ACTA2*. Dendritic cells (*CD86*, *CD80* and *CD83*), fibroblasts (*COL1A1*, *COL1A2* and *COL3A1*) and endometrial cells (*PAEP* and *DEFB1*) were identified (Figure S1).

**FIGURE 2:**
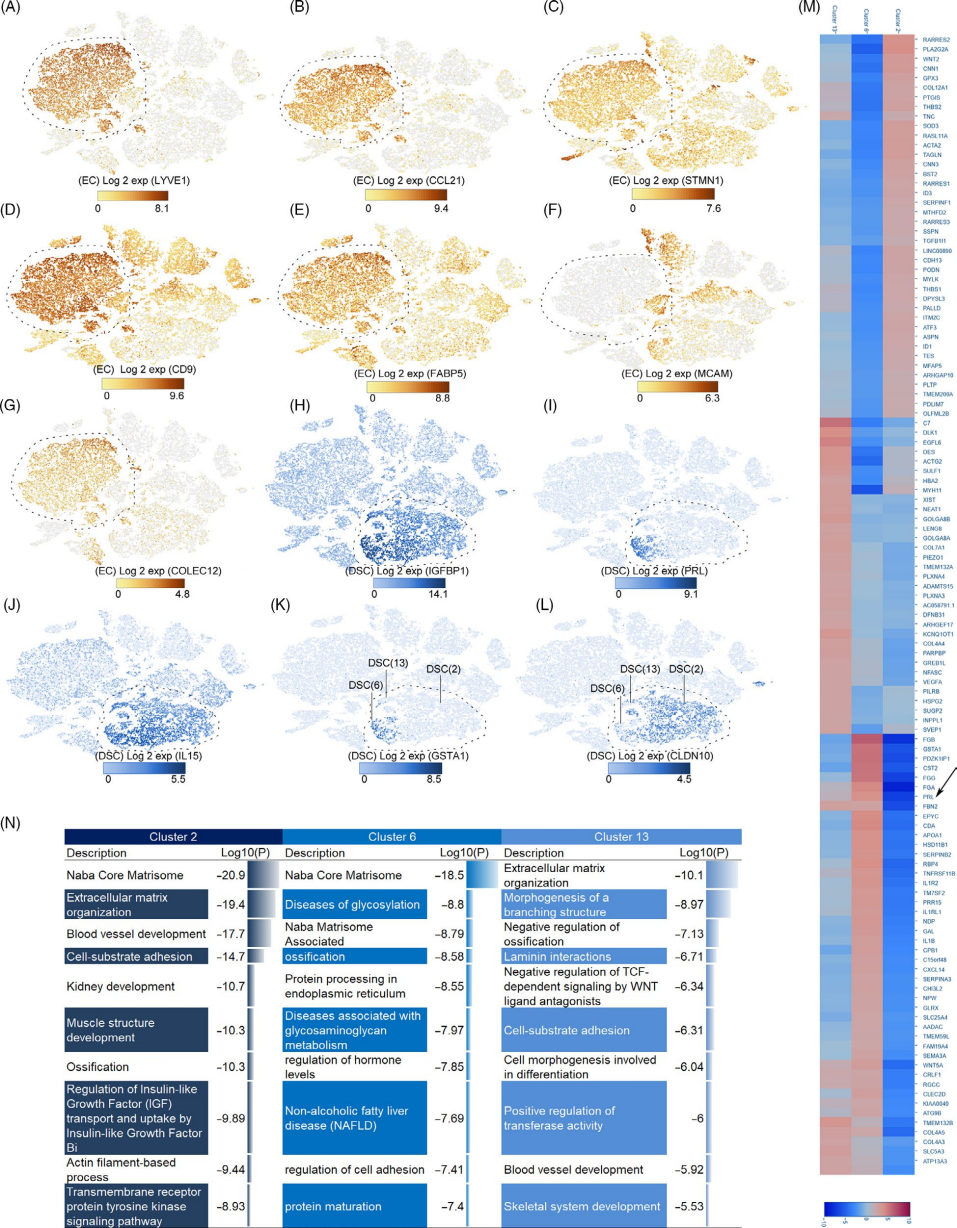
Identification of major cell populations (endothelial cell, decidual stromal cell). A‐G, Identification of EC populations. Different figure numbers represent different markers. The cell type is indicated by a dotted line. A, *LYVE1*. B, *CCL21*. C, *STMN1*. D, *CD9*. E, *FABP5*. F, *MCAM*. G, *COLEC12*. H‐L, Identification of DSC group and subgroups. H, *IGFBP1*. I, *PRL*. J, *IL‐15*. K, *GSTA1*. L, *CLDN10*. M, Heat map of gene expression among three subgroups of DSCs. The arrow indicates differential expression of *PRL* in subgroups. N, Comparison of the functions of the three DSC subgroups; Log10(P) is the *P*‐value in log base 10. DSC, decidual stromal cell; EC, endothelial cell

We observed two endothelial cell populations (clusters 1 and 3), which expressed *LYVE1, CCL21, STMN1, CD9* and *FABP5* (Figure 2A‐E).^14^ During vascular development, ECs are the first units formed, and a vascular network is formed on the basis of these cells. Generally, endothelial cells are classified into different types such as vascular endothelium cells and lymphatic endothelium cells depending on their location. Vascular endothelium cells and lymphatic endothelium cells are homologous and have similar expression characteristics. In the process of vascular development, both vascular endothelium cell and lymphatic endothelium cell markers may exist temporarily in the same cell. Using more precise markers (*MCAM* for vascular endothelium cells and *COLEC12* for lymphatic endothelium cells),^22^ vascular endothelium cells and lymphatic endothelium cells were found distributed in both clusters 1 and 3 (Figure 2F,G).

As one of the best indicators of decidualization,^23^ *IGFBP1* can be used to recognize decidual stromal cells in peripartum decidua (Figure 2H). *PRL* and *IL‐15* were differentially expressed in decidual stromal cells (Figure 2I,J). Consistent with this result, subpopulations with low *PRL* expression in decidual stromal cells were found during early pregnancy.^13^ In addition to the inconsistent *PRL* expression in these three subgroups (cluster 2, cluster 6 and cluster 13), *GSTA1* and *CLDN10* expression differences could be used to distinguish them (Figure 2K,L). Cluster 6 showed high expression of *GSTA1* specifically, while only cluster 13 showed low expression of *GSTA1* and *CLDN10* (Figure 2K,L).

We analysed the expression of genes found specifically in the decidual stromal cell population and found that the main functions of decidual stromal cells at this stage included extracellular matrix organization, protein processing and cell‐substrate adhesion. Through single‐cell RNA sequencing, we also found that although decidual stromal cells may perform the above functions, there still exists intra‐group heterogeneity (Figure 2M,N). In addition to extracellular matrix organization and cell‐substrate adhesion, the characteristic genes expressed by cluster 2 were found to be mainly involved in regulation of insulin‐like growth factor (*IGF*) transport and muscle structure development (Figure 2N). The difference was that cluster 6 genes focused on processes including protein processing in the endoplasmic reticulum and regulation of hormone levels. We also noted that this cluster expressed a higher level of classical decidual stromal cell markers (*IGFBP1* and *PRL*), indicating the importance of these genes in decidualization (Figure 2H,I,M,N).

**FIGURE 3:**
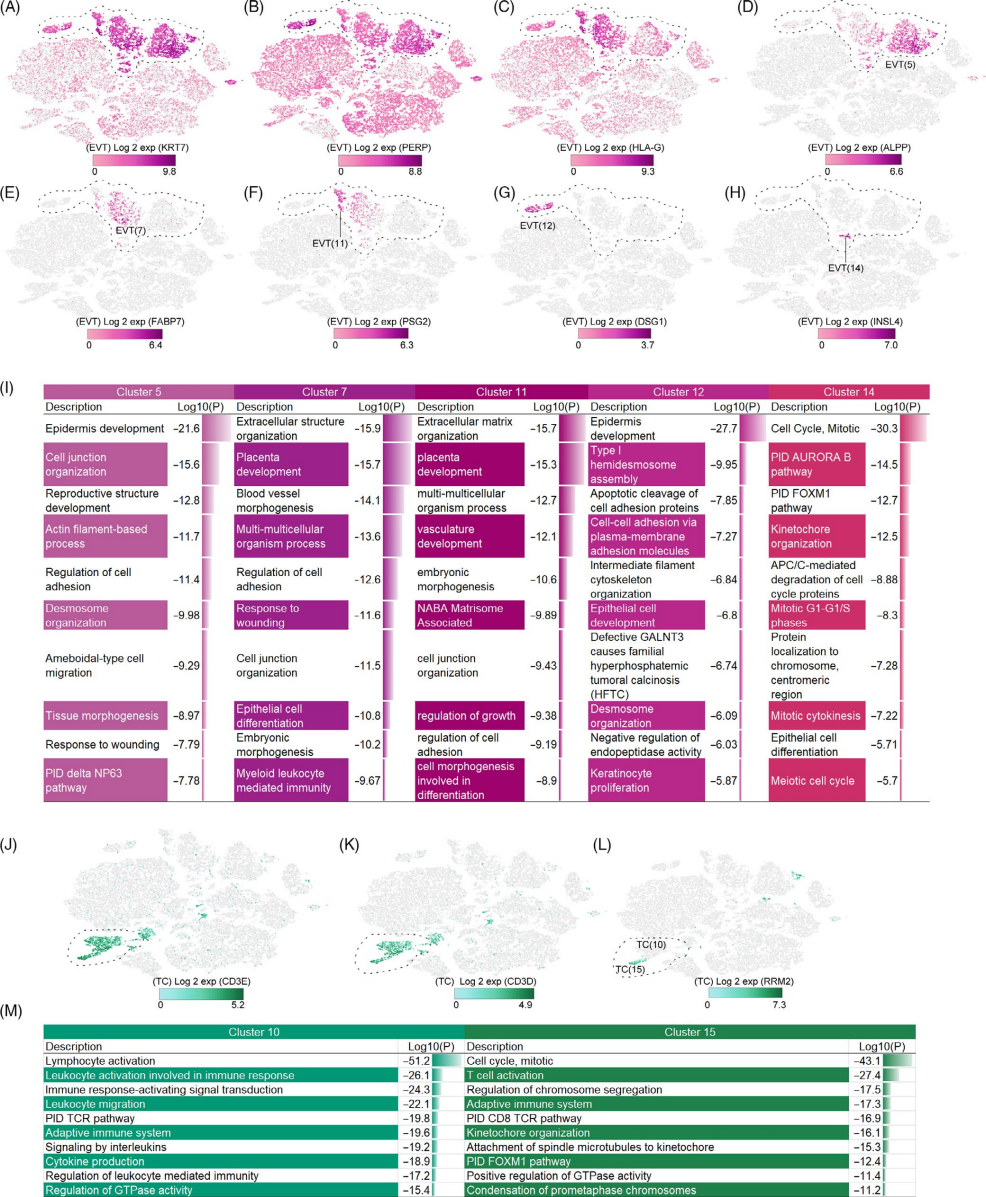
Identification of major cell populations (extravillous trophoblast, T cell). The cell type is indicated by a dotted line. A‐C, Identification of EVT group. A, *KRT7*. B, *PERP*. C, *HLA‐G*. D‐H, Identification of EVT subgroup. D, *ALPP*. E, *FABP7*. F, *PSG2*. G, *DSG1*. H, *INSL4*. I, Comparison of the functions of the EVT subgroups; Log10(P) is the *P*‐value in log base 10. J, K, Identification of TC group. J, *CD3E*. K, *CD3D*. L, Identification of TC subgroup (*RRM2*). M, Comparison of the functions of the TC subgroups; Log10(P) is the *P*‐value in log base 10. EVT, extravillous trophoblast; TC, T cell

Extravillous trophoblasts (cluster 5, cluster 7, cluster 11, cluster 12 and cluster 14) specifically expressed *KRT7*, *PERP* and *HLA‐G* (Figure 3A‐C), and the expression of *HLA‐G* differed in each subgroup (Figure 3C). The cluster 7 subgroup had the highest expression, which suggested that extravillous trophoblasts may be involved in the process of trophoblast invasion or maternal‐foetal immune tolerance in an unbalanced state.^24^, ^25^ The embryonic development function (*ADM*, *ASCL2*, *CDKN1C*, *CEBPB*, *CTNNB1*, *EFNA1*, *EGFR*, *EPAS1*, *FBN1*, *FBN2*) suggested extravillous trophoblasts had a foetal source. Using our data, we found five extravillous trophoblast subgroups with different markers: cluster 5 highly expressed *ALPP*, cluster 7 highly expressed *FABP7*, and cluster 12 highly expressed *DSG1* (Figure 3D‐H).

Trophoblasts regulate invasive functions such as neutrophil activation and cell adhesion. The high heterogeneity of extravillous trophoblasts may be one of the reasons for its multiple functions. Specifically, the top gene expressed by cluster 5 indicated that this cluster might be involved in reproductive structure development and epidermal development (Figure 3I). The main function of cluster 11 (placenta development, vasculature development) indicated that this cluster was primarily involved in the establishment and development of placenta. Considering the gestational week (more than 37 weeks) of the samples, this suggested that this group may be involved in placenta formation from the first to third trimesters. In particular, as the smallest extravillous trophoblast group, cluster 14 has mainly two functions, DNA replication and cell division, indicating that this was a subgroup of cell proliferation (Figure 3I).

We can identify the T‐cell group based on the expression of *CD3* (*CD3E*) and *CD3D* (Figure 3J,K). We found that T cells had two sets of clusters, which were not strictly classified in the classical way (eg, CD4+ TC and CD8+ TC), but had unique transcriptome differences. The two subgroups (clusters 10 and 15) of T cells can be distinguished by *RRM2* (cluster 15+ and cluster 10−; Figure 3L).

As immune cells, the main functions of T cells are lymphocyte activation and the adaptive immune response. Cluster 10 focused on T‐cell activation, CD8+ T‐cell receptor response pathways, and may be a cell population that primarily functions in the immune response (Figure 3M). We observed that cluster 15 cells were significantly different from those of cluster 10, which consisted of a significant proportion of cells at various stages of the cell cycle, including mitosis, presumably related to T‐cell proliferation (Figure 3M).

### 3.2 Change in cell number and proportion in the decidua during the peripartum period

**FIGURE 4:**
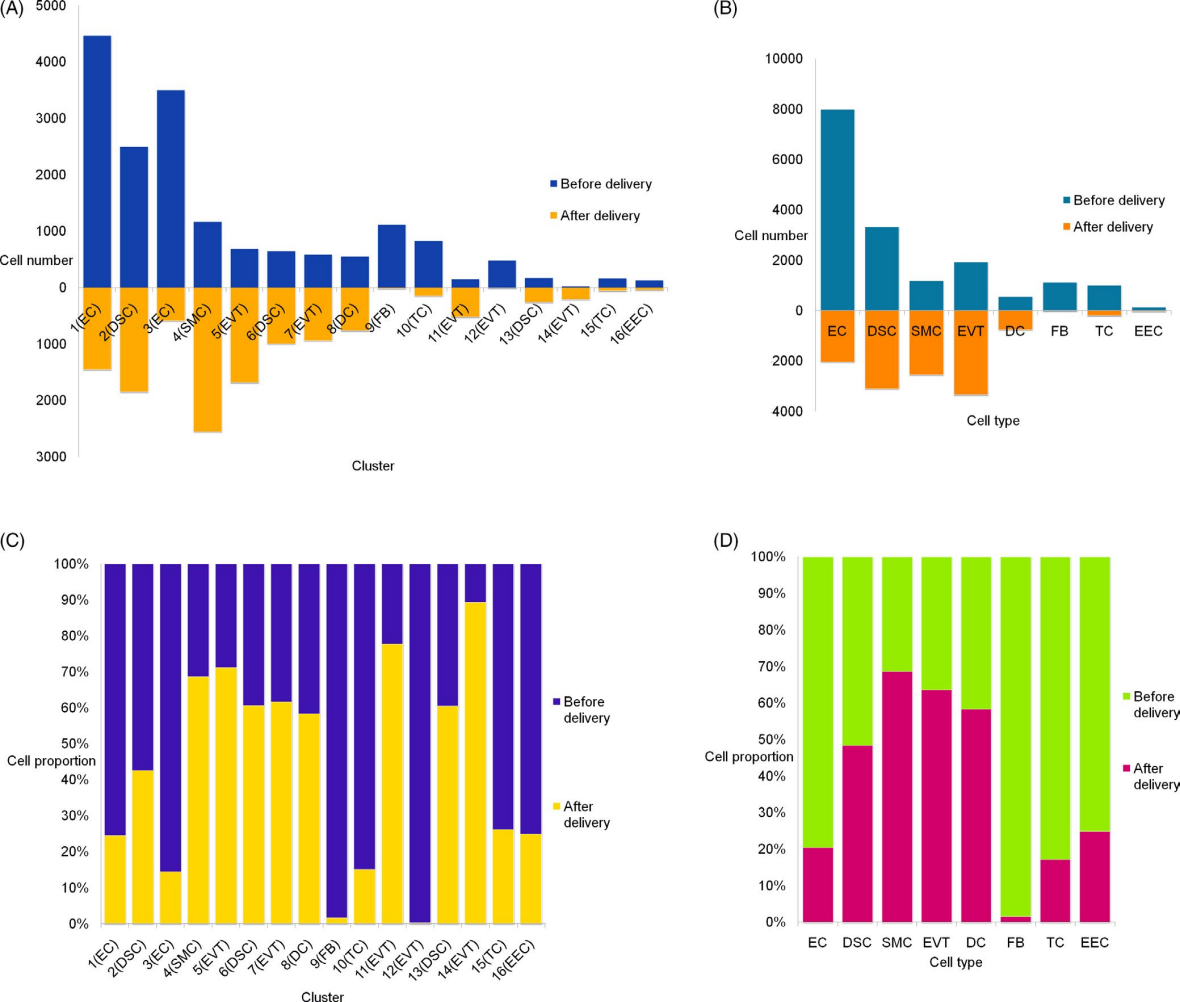
Cell number and proportions of decidua during peripartum. A, Comparison of the cell numbers in each cluster before and after delivery. B, Comparison of the cell numbers in each cell type before and after delivery. C, Comparison of the cell proportions in each cluster before and after delivery. D, Comparison of the cell proportions in each cell type before and after delivery. DC, dendritic cell; DSC, decidual stromal cell; EC, endothelial cell; EEC, endometrial cell; EVT, extravillous trophoblast; FB, fibroblasts; SMC, smooth muscle cell; TC, T cell

Analysis of 29 231 cells (eight types, 16 clusters) in our study showed that the top three cell types were endothelial cell (10 004, 34.22%), decidual stromal cell (6422, 21.97%) and smooth muscle cell (3720, 12.73%; Figure 1B, Table S2). In the first trimester, decidual leucocytes accounted for more than 40% of all cells; endothelial cells were few in number.^14^ During the peripartum period, the proportion of decidual endothelial cells increased greatly and that of fibroblasts decreased. The proportional change in decidual stromal cell numbers was relatively small. Compared to the first‐trimester data,^14^ the proportions of certain cell types varied greatly during the peripartum period (Figures 1B and 4, Table S2).

The largest group was cluster 1 (endothelial cell, 5919, 20.25%), followed by cluster 2 (decidual stromal cell, 4345, 14.86%), cluster 3 (endothelial cell, 4085, 13.97%) and finally cluster 16 (endometrial cell, 169, 0.58%), which was the smallest (Figure 1B, Table S2). We found that the order of the number of cells in each cluster after delivery was inconsistent with that before delivery. After labour onset, the largest proportion was cluster 4 (smooth muscle cell, 2553, 21.13%), followed by cluster 2 (decidual stromal cell, 1849, 15.30%) and cluster 5 (extravillous trophoblast, 1684, 13.94%) (Figure 4). Our results suggested that although the cell type was relatively stable during this period, the number of cells changed, especially smooth muscle cells, which may be active during labour onset. Therefore, in terms of cell number, smooth muscle cells were likely the main effector cells during labour and the main active cell type in the decidua. Overall, our results suggest there is heterogeneity in terms of cell numbers and proportions in the decidua.

### 3.3 Heterogeneity of expression profiles of different cell types during the peripartum period

Next, we analysed transcriptome changes during labour onset. We found that not only did the expression profiles of different cell types vary unequally, but also one of the subtypes was also inconsistent. The genes that were upregulated by most cells reflected the functions they performed during this period. For example, smooth muscle cells were the most active cell population after delivery, and the upregulated genes (TOP: *CSF3*, *CXCL1*, *DIO3*, *IL24*, *CHI3L1*, *IL6*, *CXCL2*, *CHI3L2*, *NR4A1*, *CXCL8*) were involved in the regulation of SMC proliferation, suggesting adaptability of cell function and cell status.

**FIGURE 5:**
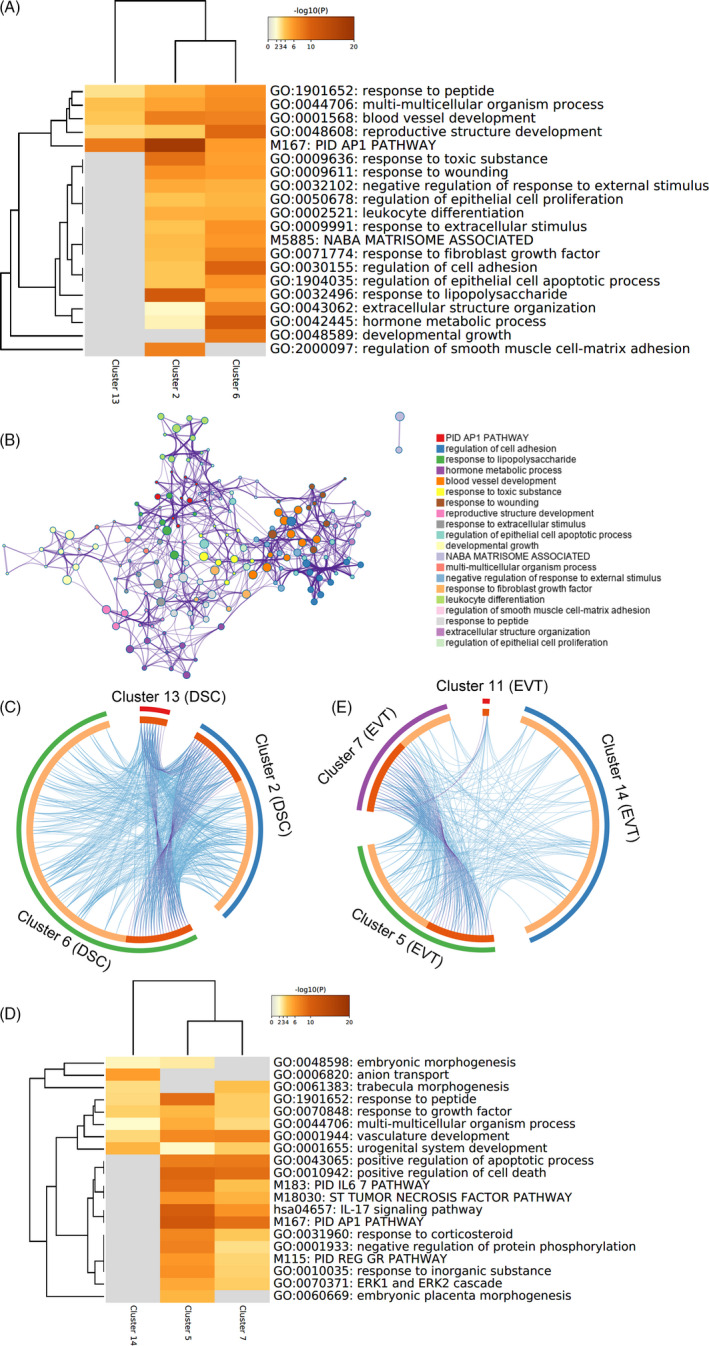
Comparison of the functions of upregulated genes among some cell populations after delivery. A, Heat map of enriched terms across upregulated genes of decidual stromal cell subgroups after delivery. Log10(P) is the *P*‐value in log base 10. B, Network of enriched terms in decidual stromal cell subgroups, coloured by cluster ID, where nodes that share the same cluster ID are typically close to each other. C, Overlap between genes of decidual stromal cell subgroups, including the shared term level, where blue curves link genes that belong to the same enriched ontology term. The inner circle represents gene lists, where hits are arranged along the arc. Genes that hit multiple lists are coloured in dark orange, and genes unique to a list are shown in light orange. D, Heat map of enriched terms across upregulated genes of extravillous trophoblast subgroups after delivery. After delivery, the other two clusters had no significantly upregulated genes or the number of upregulated genes was not sufficient for functional analysis. Log10(P) is the *P*‐value in log base 10. E, Overlap between genes of extravillous trophoblast subgroups, including the shared term level, where blue curves link genes that belong to the same enriched ontology term. The inner circle represents gene lists, where hits are arranged along the arc. Genes that hit multiple lists are coloured in dark orange, and genes unique to a list are shown in light orange. Cluster 12 of extravillous trophoblast had no significant upregulated gene after delivery. DSC, decidual stromal cell; EVT, extravillous trophoblast

In the decidual stromal cell group, we compared the gene upregulation levels among the three subgroups (clusters). The identified genes were principally involved in the response to lipopolysaccharide, and activation of the activator protein 1 pathway (Figure 5A). Cluster 2 had only 66 genes significantly upregulated; these genes reflect roles in the activator protein 1 pathway, response to lipopolysaccharide and regulation of smooth muscle cell‐matrix adhesion. The heat map and ring map visually show the interrelationships among these subgroups (Figure 5A‐C). As shown, the main active subgroups in this process are clusters 2 and 6, which perform similar functions.

The expression profile of cluster 12 had no significant change after labour, suggesting that this may be a ‘stable’ cluster. Clusters 5 and 7 were more active in this process and have a closer relationship with each other (Figure 5D‐E).

**FIGURE 6:**
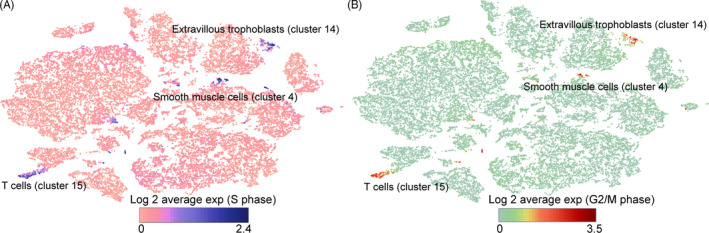
The expression of cell cycle–related genes in decidual cell population. A, S phase–related cell‐cycle gene expression. B, G2/M phase–related cell‐cycle gene expression

Our results showed that the functions of T cells were lymphocyte activation and the adaptive immune response during this period. Cluster 10 mainly performed these primary functions (Figure S2). Unexpectedly, the results of cluster 15 were significantly different from that of cluster 10, which consisted primarily of cells in mitosis and the cell cycle (Figure 6 and Figure S2). We speculated that this phenomenon might be related to T‐cell proliferation. Although the role of decidual immune cells in the maternal‐foetal interface has not been fully elucidated, our results suggest that heterogeneity exists in the T‐cell population.

### 3.4 Differences in cell cycle and proliferation in perinatal decidual tissue

To evaluate cell‐cycle differences in perinatal decidual tissue, we analysed the expression of cell‐cycle genes.^26^

Cells in S phase were mainly concentrated in clusters 4 (a small part of smooth muscle cell), 14 and 15 (Figure 6A). These clusters (cluster 4 smooth muscle cell, cluster 14 extravillous trophoblast and cluster 15 T cell) also highly expressed G2/M phase cell‐cycle genes (Figure 6B). These results suggest that cells in these clusters may have shorter cell cycles or more active proliferation. Compared to other clusters, the cells in these three clusters were more proliferative and may not be affected by labour. During pregnancy, the immune system is functional and highly active.^27^ Among active decidual cells, T cells make up the largest proportion, suggesting a high degree of immune cell proliferation during the perinatal period.

### 3.5 Pseudotemporal ordering reveals a differentiation relationship between major cell types

**FIGURE 7:**
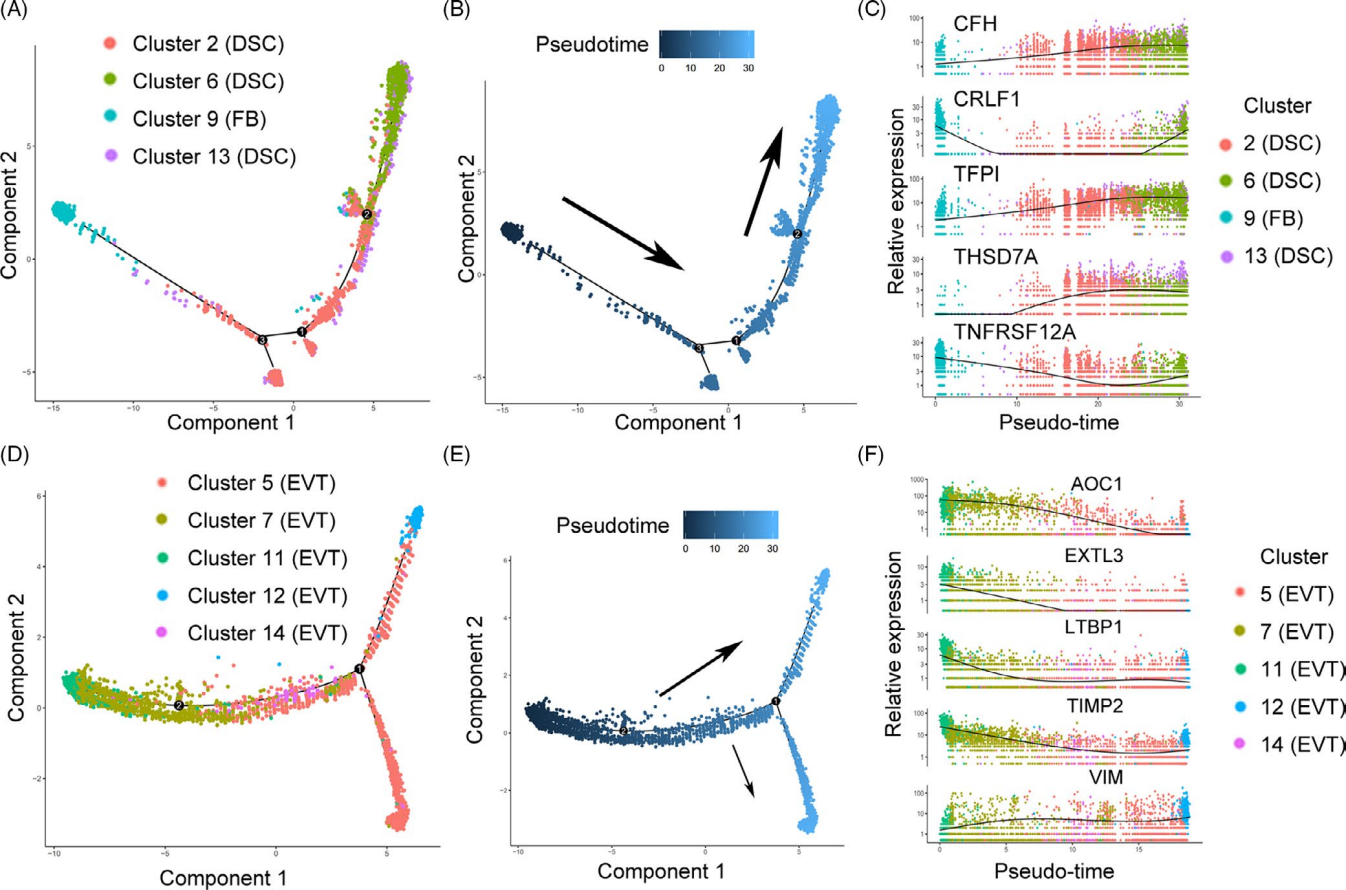
Pseudotemporal ordering of decidual cells. A, Pseudotemporal ordering of cluster 2 (DSC), cluster 6 (DSC), cluster 9 (FB) and cluster 13 (DSC). The numbers inside the black circles represent the different cell status numbers identified in the trajectory analysis. B, Pseudotemporal ordering trajectory map (cluster 2, cluster 6, cluster 13 and cluster 9). The colours from dark to light represent the order of pseudo‐time. C, Differential gene pseudotemporal expression trajectory map (cluster 2, cluster 6, cluster 13 and cluster 9). D, Pseudotemporal ordering of (EVT) cluster 5, cluster 7, cluster 11, cluster 12 and cluster 14. The numbers inside the black circles represent the different cell status numbers identified in the trajectory analysis. E, Pseudotemporal ordering trajectory map (EVT cluster 5, cluster 7, cluster 11, cluster 12 and cluster 14). F, Differential gene pseudotemporal expression trajectory map (EVT cluster 5, cluster 7, cluster 11, cluster 12 and cluster 14). DSC, decidual stromal cell; EVT, extravillous trophoblast; FB, fibroblasts

**FIGURE 8:**
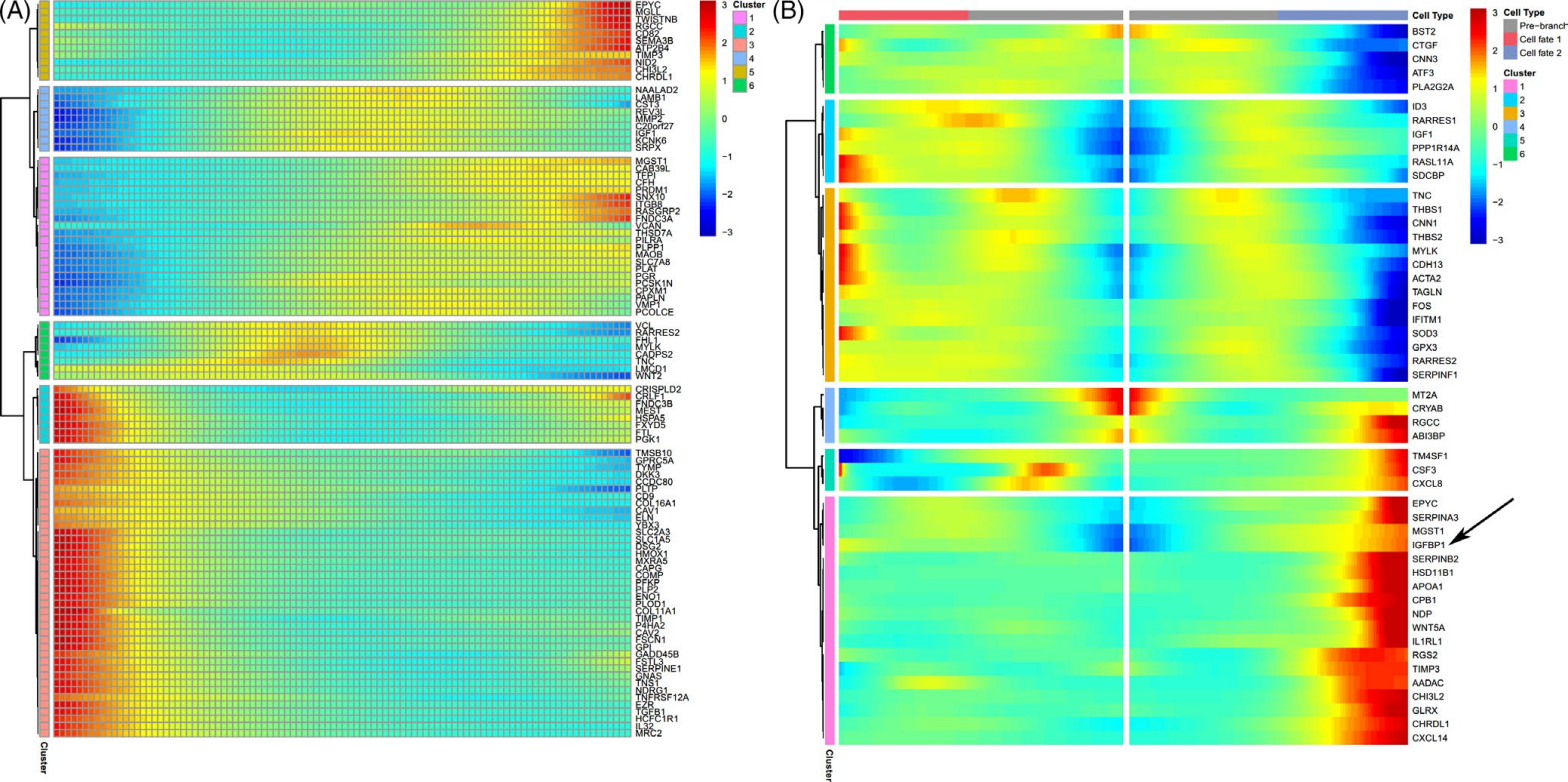
Differential gene expression (DSC clusters 2, 6 and 13; and FB cluster 9) heat map of pseudotemporal ordering in decidua. A, Differential gene cluster heat map (for DSC clusters 2, 6 and 13; and FB cluster 9). Each column represents the average expression in the current cell state. The ‘cluster’ in this figure represents a cluster of genes with similar dynamic trends, which is different from the ‘cluster’ of cells in this article. B, Differential gene pseudotemporal expression trajectory map (for DSC clusters 2, 6, and 13; and FB cluster 9). The arrow indicates the expression of IGFBP1 gradually increased along the trajectory of cell fate 2. DSC, decidual stromal cell; FB, fibroblasts

Next, we performed pseudotemporal ordering of decidual stromal cells (Figure S3A‐C). Our results showed an evolutionary trajectory of decidual stromal cell subtypes and a differentiation relationship with fibroblasts (Figure 7A,B). We found a differentiation relationship from cluster 2 to cluster 6 by decidual stromal cell pseudotemporal ordering and believe cluster 6 may be at the end of differentiation (Figure 7A,B and Figure S3A,B). When FBs were included in our analysis, the new trajectory was the direction from fibroblast to decidual stromal cell (Figure 7A,B). Along this trajectory, the expression of *CFH* and TFPI gradually increased (Figure 7C). We divided the genes into 6 ‘clusters’ to classify genes that followed similar dynamic trends in the trajectory, and found that the expression of most genes gradually decreased (Figure 8A). In ‘cluster’ 3 of Figure 8A, the decreasing genes were involved in processes including extracellular structure organization, inflammatory response and cell junction organization. This change reflected the process of functional evolution or the change from fibroblast to decidual stromal cell. An analysis of branch events revealed differential genes associated with branches (Figure 8B). We found that *IGFBP1* (Figure 8B) was one of the key genes for cell fate 2, which was related to the decidual stromal cell fate.

Among extravillous trophoblasts, cluster 11 to cluster 12 followed a similar pseudotemporal ordering (Figure 7D,E and Figure S3D,E). These results suggest the associations among these subgroups. Along the trajectory, the expression of *TIMP2* showed a gentle downward trend (Figure 7F). The high expression of *TIMP2* in decidual extravillous trophoblasts is important for matrix remodelling and controlled trophoblast invasion during placentation.^28^ Our results suggest that the changes in extravillous trophoblast subgroups occur during invasion during this period.

## 4 DISCUSSION

The maternal‐foetal interface is complex; the various cells involved can be identified via single‐cell sequencing. Prior single‐cell sequencing papers studied placental and decidual heterogeneity during early pregnancy.^12^, ^13^, ^14^ However, pregnancy is a period with changes, and the heterogeneity of the decidua may change during the peripartum period. Our research focused on the composition (eight types, 16 clusters) and cellular changes in the decidua in two states before and after labour. Due to the different recognition efficiency of different markers, such as endothelial cells, it was difficult to reclassify cells accurately (like lymphatic endothelium cell or vascular endothelium cell). Similar situations may exist in other cell types that have not been evaluated systematically.

Based on the results on peripartum decidual cell proportions, the main cell population of the decidua gradually changed from fibroblasts and decidual stromal cells in early pregnancy to endothelial cells and decidual stromal cells. The decrease in fibroblasts suggests this group has the capacity to differentiate. The fibroblast subpopulations in early pregnancy rely on *IGF1* regulation in the decidual microenvironment. The low expression of *IGF1* in the fibroblast group suggests that fibroblasts may also be related to the differentiation of cells in the fibroblast subgroup.^14^ Next, we performed in‐depth cell function analyses to gain further insight. In the active state of delivery, fibroblasts participated in vascular morphogenesis, which suggests that fibroblasts may be involved in angiogenesis (endothelial cell). Other cells also have the ability to differentiate into endothelial cells that may be associated with inflammation‐induced preterm birth.^29^, ^30^, ^31^ At the single‐cell RNA‐sequencing level, these results suggest the existence of vascular remodelling within decidual tissue, which provides structural and functional supports for the rapid changes that occur during labour.

The function of cells may be in accordance with their proportion. For example, the endothelial cells of early pregnancy engage in (principally) angiogenesis and vascular remodelling.^32^ At that stage, the proportion of decidual endothelial cells is only 6%.^14^ Our peripartum data revealed that the proportion of endothelial cells exceeded 30%, thus greater than the level evident in early pregnancy. After the development and proliferation of endothelial cells in the second trimester, a large number of endothelial cells participate in the vasculature development and nutrition transport in the third trimester. These functions are important for maintaining the pregnancy and for delivery, especially during labour. During this period, the foetal oxygen demand and microenvironment change, and delivery‐related cytokines are released. These processes require an interactive environment composed of a large number of endothelial cells; therefore, the change in the proportion of decidual cells may be accompanied by a functional change, which provides the basis for performing the required actions.

In addition, our study analysed the relationship between decidual extravillous trophoblasts and labour onset using single‐cell RNA sequencing. High expression of *HLA‐G* on the cell surface of actively migrating extravillous trophoblasts is also related to extravillous trophoblast invasion in early pregnancy and interaction with natural killer cells.^24^, ^25^, ^33^, ^34^, ^35^, ^36^ *HLA‐G* is also involved in maternal‐foetal interface immune tolerance, including uterine immune cell activation and remodelling.^34^, ^37^ In our results, cluster 11 (extravillous trophoblast) expressed the highest levels of *HLA‐G*, suggesting that the extravillous trophoblast subgroups did not equally participate in the aforementioned functions. Furthermore, the functions of the active extravillous trophoblast group focused on cell death regulation and vasculature development, involving interleukin‐17, activator protein 1 and interleukin‐6 signalling pathways. It is worth noting that the role of activator protein 1 and interleukin‐6 signalling in labour has been confirmed.^38^, ^39^, ^40^, ^41^ As one of the extravillous trophoblast subgroups (cluster 12) was not active during labour, its function in delivery is unclear. These results suggest that extravillous trophoblasts may participate in labour by regulating immune tolerance and the activation of multiple signalling pathways. There is no doubt that the participation of various extravillous trophoblast subtypes in the initiation of labour onset is a complex process that warrants needs further investigation.

Among decidual stromal cells, which are characteristic decidual cells, we found that clusters 2 and 6 were mainly involved in labour onset. In general, these subgroups were involved in the activator protein 1 pathway, suggesting that decidual stromal cells in delivery may be regulated through this pathway. This pathway is also related to the activation of smooth muscle cells.^42^ Although smooth muscle cells are currently recognized as effectors during delivery, the exact mechanism is still unclear.^43^ Further research may solve the problem of intercellular communication of decidual stromal cell‐smooth muscle cell and explain the role of decidual stromal cell‐smooth muscle cell activation in delivery.

Extravillous trophoblasts with high expression of *HLA‐G* are related to immune tolerance, and in the perinatal period, the expression profile of decidual T cells that regulate immunity is heterogeneous. Our results showed that most T cells perform immune responses, while a small number retain their ability to proliferate. In normal pregnancy, early decidual CD8+ T cells have both activation and dysfunction.^44^ As pregnancy progresses, the reactivity of maternal‐foetal antigen‐antibody gradually changes and adapts to each other. We discovered differences in the proportions of different functional subgroups of decidual T cells. This difference may be closely related to the foetal‐maternal immune tolerance response during labour onset. During pregnancy, T cells maintain the maternal‐foetal tolerance, thus a normal pregnancy.^45^ Decidual T‐cell numbers change during delivery, perhaps in an effort to maintain homeostasis.^46^ Dendritic cells regulate T‐cell responses; T cells interact via a complex web during pregnancy.^47^ Decidual immune cells may thus play important roles in pregnancy maintenance and labour onset.

Data on early pregnancy have shown the existence of pseudotemporal ordering from fibroblasts to decidual stromal cells,^14^ which is consistent with our results. Cluster 6 may be the end of the ordering (cell fate 2 in Figure 8B) in decidual stromal cells. According to the ordering, the expression change in complement factor H (which can inhibit CD47‐mediated resolution of inflammation) in the pseudotemporal ordering suggests the functional heterogeneity of decidual stromal cells.^48^ *TFPI* has been implicated in angiogenesis‐related processes^49^; this was similar to the function of cluster 6 after labour onset (Figure 5A). The data indicate that the pseudotemporal ordering might be preparation for labour onset. In the extravillous trophoblast ordering, *TIMP2* expression showed a relatively slow decreasing trend (Figure 7F), suggesting heterogeneity of extravillous trophoblast subgroups. Previous studies have shown that *TIMP2* is associated with homeostatic regulation of extracellular matrix remodelling in labour onset.^50^

We used single‐cell sequencing to study peripartum decidua. Further work is required; this is a limitation of our research. High‐level expression of cell cycle–relevant genes in certain cell populations may indicate that these cells can proliferate. However, cell‐cycle signals may mask non–cell cycle differences, which may need further study. We sampled only six pregnant women; sampling error may be in play in terms of cell type/subtype identification and RNA expression patterns. Therefore, more comprehensive researches may help to solve this issue.

In conclusion, our study used single‐cell RNA‐sequencing technology and revealed new information on the peripartum decidual atlas and cell proportions. Furthermore, we revealed heterogeneity in the subgroups and found differential gene expression and functional changes. The various cell types participate in labour to varying degrees, demonstrating the complexity of cell networks that form the decidual tissue system associated with delivery. In summary, our results provide a new perspective for the study of delivery, and provide important single cell–based evidence for detailed studies on the decidua during the peripartum period.

## CONFLICT OF INTEREST

The authors declare no conflicts of interest.

## AUTHOR CONTRIBUTIONS

J.H. and Q.L. conceptualized the data; J.H., Q.L., Q.P., L.X. and J.Z. curated the data; C.P., Y.Z., L.X. and J.Z. involved in formal analysis; J.Z. and W.Z. acquired funding; Q.P., L.D., J.C. and J.S. investigated the data; J.H., Q.L., Q.P. and Y.X. designed methodology; W.Z. administered the project; Y.X., W.W., C.P., Y.Z., R.L., L.H. and T.L. provided resources; W.Z. supervised the data; J.H. wrote the original draft; and Q.L. and W.Z. wrote, reviewed and edited the manuscript. All authors read and approved the final manuscript.

## Supporting information

Fig S1‐S3

Table S1

Table S2

## Data Availability

The data that support the findings of this study are available from the corresponding author on reasonable request.
