# Supplementary material for: Single‐cell RNA sequencing reveals heterogeneity and differential expression of decidual tissues during the peripartum period
Source: Cell Prolif. 2020 Dec 9;54(2):e12967. doi: 10.1111/cpr.12967 (PMC7848970; doi:10.1111/cpr.12967)
Supplement: Supplementary file 1 — Fig S1‐S3 [file CPR-54-e12967-s001.doc]

Single cell RNA sequencing reveals heterogeneity and differential expression of decidual tissues during the peripartum period

Supplementary Materials (Figures S1-S3)

**
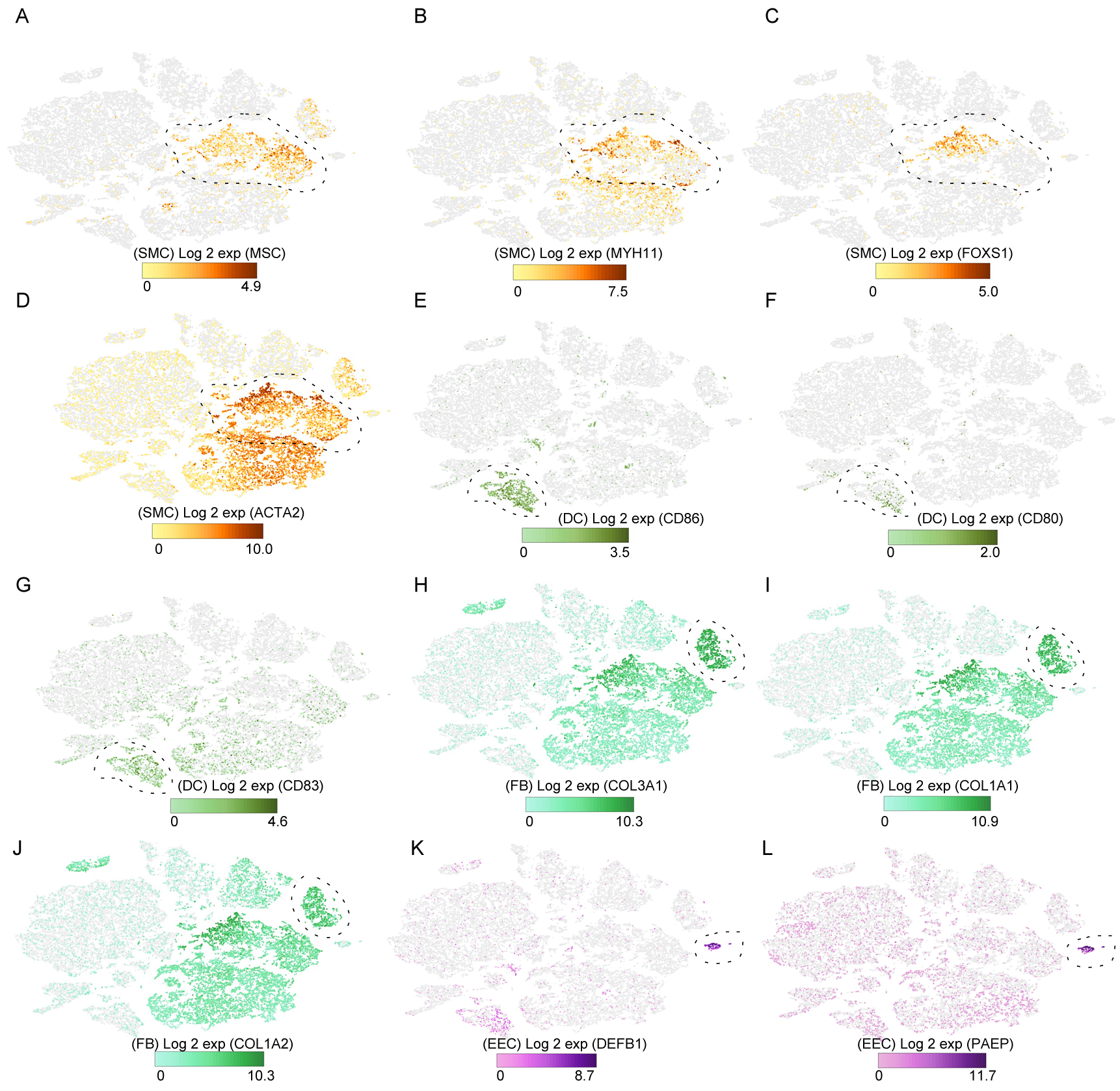
**

Figure S1. The identification of other cell populations. A-D, The identification of SMC. Different figure numbers represent different markers. A, MSC. B, MYH11. C, FOXS1. D, ACTA2. E-G, The identification of DC. Different figure numbers represent different markers. E, CD86. F, CD80. G, CD83. H-J, The identification of FB. Different figure numbers represent different markers. H, COL3A1. I, COL1A1. J, COL1A2. K, L, The identification of EEC. Different figure numbers represent different markers. K, DEFB1. L, PAEP. SMC: smooth muscle cell. DC: dendritic cell. FB: fibroblast. EEC: endometrial cell. EVT: extravillous trophoblast.


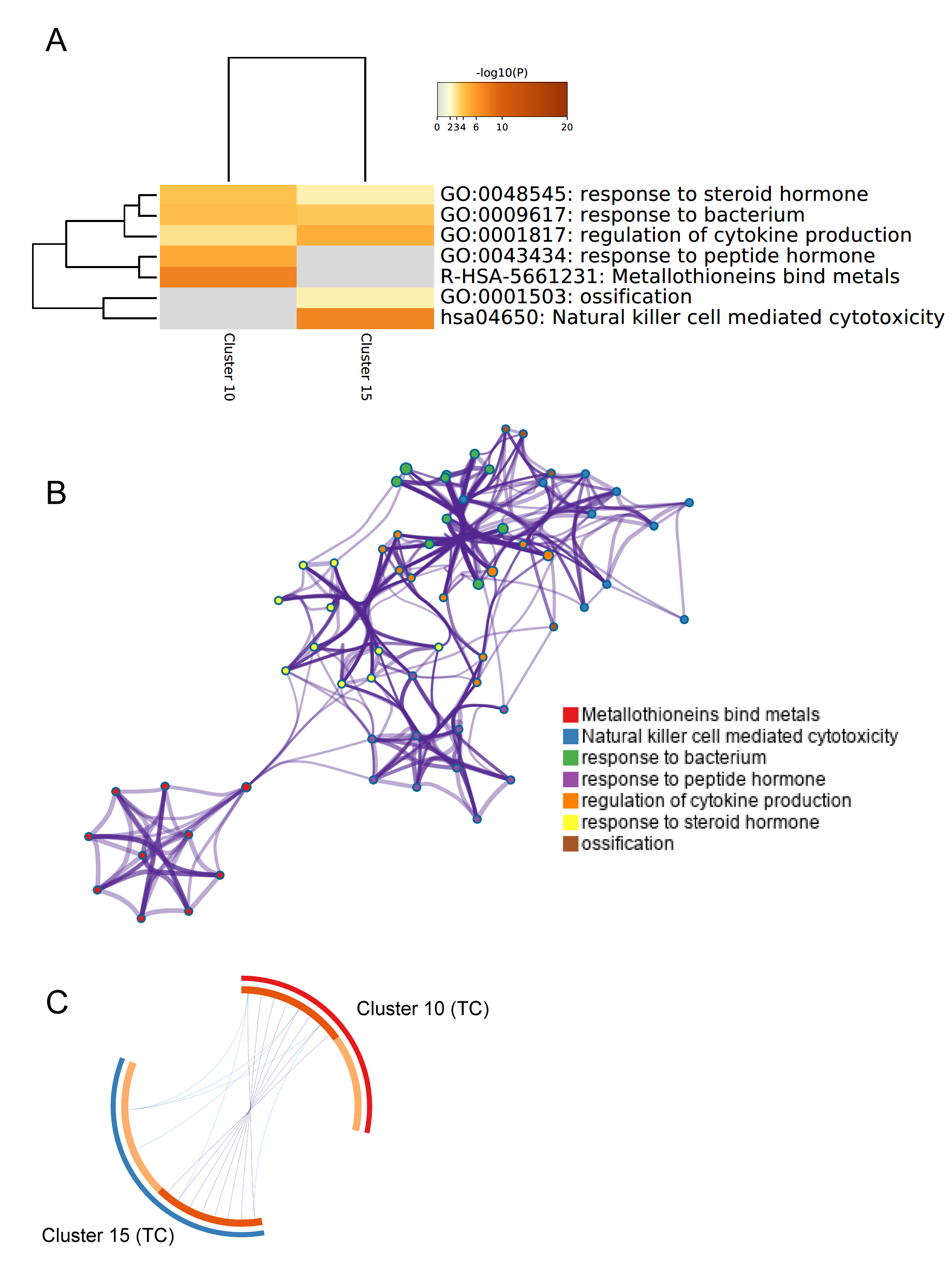


Figure S2. Comparison of functions of up-regulated gene among TCs after delivery. A, Heatmap of enriched terms across upgraded genes of TC subgroups after delivery. Log10 (P) is the p-value in log base 10. B, Overlap between gene lists of TC subgroups, including the shared term level, where blue curves link genes that belong to the same enriched ontology term. The inner circle represents gene lists, where hits are arranged along the arc. Genes that hit multiple lists are colored in dark orange, and genes unique to a list are shown in light orange. C, Network of enriched terms of TC subgroups, colored by cluster ID, where nodes that share the same cluster ID are typically close to each other. TC: T cell.


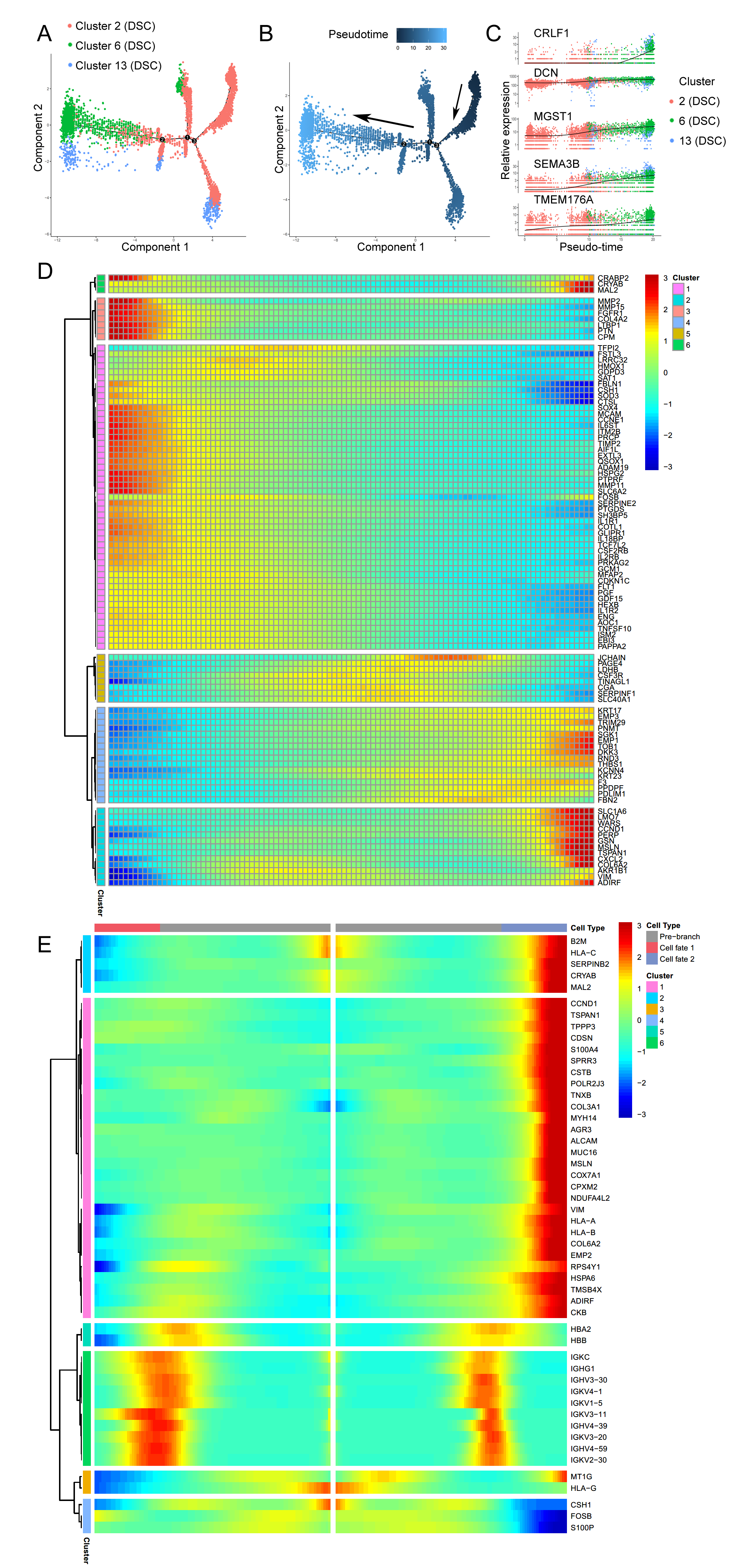


Figure S3. Pseudotemporal ordering of the DSCs and EVTs in decidua. A, Pseudotemporal ordering of cluster 2 (DSC), cluster 6 (DSC) and cluster 13 (DSC). The numbers inside the black circles represent the different cell status numbers identified in the trajectory analysis. B, Pseudotemporal ordering trajectory map (DSC, cluster 2, cluster 6, cluster 13). The colors from dark to light represent the order of pseudo-time. C, Differential gene pseudo-temporal expression trajectory map (DSC, cluster 2, cluster 6, cluster 13). D, Differential gene cluster heat map (EVT, cluster 5, cluster 7, cluster 11, cluster 12, cluster 14). Each column represents the average expression in the current cell state. The “cluster” in this figure represents a cluster of genes with similar dynamic trends, which is different from the “cluster” of cells in this article. E, Analysis of cell data (EVT, cluster 5, cluster 7, cluster 11, cluster 12, cluster 14) at pseudo-time nodes with branched expression analysis modeling (BEAM). The “cluster” on the left in the figure is the classification of 50 differential genes, which is different from the “cluster” of cells in this article. DSC: Decidual stromal cell. EVT: Extravillous trophoblast.
